# Editorial: *Lawsonia intracellularis*: a problem well understood is a problem half solved

**DOI:** 10.3389/fvets.2023.1203702

**Published:** 2023-05-02

**Authors:** Anbu Kumar Karuppannan

**Affiliations:** Department of Veterinary Microbiology, Bioinformatics Centre, Madras Veterinary College, Tamil Nadu Veterinary and Animal Sciences University, Chennai, India

**Keywords:** *Lawsonia intracellularis*, vaccine, pathogenesis, microbiome, microbiota, genome

Porcine proliferative enteropathy (PE) or proliferative hemorrhagic enteritis (PHE), or commonly “ileitis”, is a severe intestinal disease of domestic pigs and wild boars. It is caused by the bacterium *Lawsonia intracellularis*, which infects the cells lining the ileum and infrequently cells in the large intestine, and it causes inflammation, hemorrhages, and thickening of the intestinal wall (1). The disease can be fatal, particularly in young pigs, and causes significant economic loss (2). *L. intracellularis* also infects and causes pathogenesis in horses, rabbits, and hamsters (1). The etiological role of *L. intracellularis* in ileitis was unraveled after much effort, due to the initial difficulty in the axenic culture of the organism, by Lawson et al. at the University of Edinburgh (3). *L. intracellularis* is an obligate intracellular microaerophilic organism that requires a reduced O_2_ concentration of 8%. The establishment of culture procedures opened avenues for further research on *L. intracellularis* (4). Now, the infection has been recorded in many host species (1, 5). The estimation of genome copies of the organism shed in the feces by quantitative PCR has been a reliable indicator of the infection load. The *L. intracellularis* strains are thought to have developed host species-specific adaptation, as strains derived from one host species show decreased pathogenicity in experimental cross-species infections (5). A multi-locus variable number tandem repeat (VNTR) analysis based on four sets of primers has been reported for discriminating porcine and equine isolates (6). A cross-sectional study by Yeh et al. reported in this collection, from South Korea, estimated a seropositivity of 12.3% to *L. intracellularis* in farmed rabbits in 163 farms. The study detected *L. intracellularis* genomic DNA from rabbit rectal swabs in 3.8% of rabbit farms. The presence of pigs or horses in or near the rabbit farm was a risk factor for seropositivity, indicating the potential cross-species spread of infection. The first commercial live attenuated *L. intracellularis* vaccine for pigs administered orally was available in 2001, and an inactivated parenteral vaccine was available from 2015 (1). The administration of the oral live attenuated vaccine requires a 7-day window free of antibiotic administration to pigs. In addition, interference due to maternal antibodies in the gut of piglets is a concern with the live attenuated vaccine (7). The vaccines have reduced the disease burden of *L. intracellularis* infection in pigs. Interestingly, an experimental infection, by Hansen et al. with porcine circovirus 2 (PCV2) prior to a combined *L. intracellularis*/PCV2 infection mitigated the clinical signs, lesions, and level of shedding of *L. intracellularis* infection. Prior PCV2 exposure also led to higher *L. intracellularis* antibody titers after the combined infection, which warrants further research into the cross-talk between the immune response to viral infections and *L. intracellularis* infection. The microbiota of the pig's ileum is known to influence the susceptibility to *L. intracellularis*, and the infection also adversely affects the gut microbiota (8, 9). In this Research Topic collection, Leite et al. have elaborated dysbiosis in the small and large intestine caused during *L. intracellularis* infection by analyzing the gut microbiome (microbiota). Particularly, an increase of pathobionts, including *Collinsella, Campylobacter, Chlamydia*, and *Fusobacterium*, was observed in the gut during *L. intracellularis* infection. Interestingly, the live attenuated *L. intracellularis* vaccine, in addition to decreasing the *L. intracellularis* shedding, significantly decreased the relative abundance of pathobionts, highlighting the role of the broader gut microbiome composition in the pathogenesis caused by *L. intracellularis*. Another article in this collection by Hankel et al., which is based on their analysis of fecal microbiota in pigs naturally infected with *L. intracellularis* that were varying in their vaccination status (with live attenuated oral vaccine) and clinical conspicuity, found the influence of the microbiota on the severity of disease. The causal role of the microbiota in the pathogenesis of *L. intracellularis* infection is yet to be completely unraveled.

The reference genome of *L. intracellularis* consists of a 1.46 Mb chromosome and three plasmids of 0.03, 0.04, and 0.19 Mb (NCBI Refseq IDs NC_020127 to NC_020130). A complete genome dataset of at least nine strains of *L. intracellularis* from around the world [US (PHE/MN1-00, E40504), UK (N343, LR189), Japan (Ni/JPN, Ib2/JPN, Fu/JPN), South Korea (CBNU010), and China (PPE-GX01-2022)] are available in the public domain at the end of March 2023. Notably, the E40504 strain was isolated from a horse. The genome data have led to the development of several semi-quantitative and quantitative PCR assays for the diagnosis of *L. intracellularis*. Xiao et al. expressed the outer membrane protein (Omp2) of *L. intracellularis* from a synthetic gene based on sequence information. Monoclonal antibodies specific to Omp2 were produced and found to be efficient in detecting *L. intracellularis* in Western blots, immunohistochemistry, and immunofluorescence assays. Moreover, a comparison of the gene expression profiles of the pathogenic low-passage and non-pathogenic high-passage isolate of *L. intracellularis* revealed that there were only 401 genes expressed by the pathogenic bacteria (10). Notably, one of the plasmids (A) was entirely repressed in the non-pathogenic bacteria. Transcriptomic profiling of the host ileum tissue in *L. intracellularis*-infected pigs revealed the molecular signature of cellular proliferation and inflammation (11). The stimulation of Notch-1 signaling and inhibition of the β-catenin/Wnt pathway in ileum crypt cells during the peak of infection by *L. intracellularis* has been implicated in the molecular pathogenesis (12). In addition, the apoptosis and autophagy pathways in the ileum cells were found to be dysregulated (12, 13). These altered signaling and cellular mechanisms are thought to influence the inhibition of goblet cell maturation and enhance crypt cell proliferation, leading to decreased mucin secretion and thickening of the ileum wall in *L. intracellularis* infection. The genome of *L. intracellularis* encodes for a type III secretion system (T3SS), which is commonly found in pathogenic bacteria to secrete effector proteins into host cells. Through a yeast expression array expressing hypothetical genes from *L. intracellularis*, it has been identified that LI1035 interacts with the mitogen-activated protein kinase (MAPK) pathway and regulates actin organization in yeast (14). Pereira et al. reported in this collection on the role of clathrin in the endocytosis of *L. intracellularis* into cultured porcine intestinal epithelial cells (IPEC-J2). They found that *L. intracellularis* is endocytosed by both clathrin-dependent and -independent mechanisms, but the internalization of heat-killed bacteria is decreased in the clathrin-depleted cells. This suggests potential active mechanisms, involving a secretion apparatus, effected by *L. intracellularis* during endocytosis, as observed in many pathogenic intracellular bacteria. Given the wealth of knowledge on the genome of *L. intracellularis*, potential effector proteins or virulence factors remain to be characterized. With the current level of information on the biology of *L. intracellularis*, a promising outlook prevails over the efforts for a comprehensive understanding of the pathogenesis of this fastidious organism, which may require uncharacterized inciting factors to cause disease.

## Author contributions

The author confirms being the sole contributor of this work and has approved it for publication.

## References

[1] Karuppannan AK Opriessnig T. *Lawsonia intracellularis*: revisiting the disease ecology and control of this fastidious pathogen in Pigs. Front Vet Sci. (2018) 5:181. 10.3389/fvets.2018.00181 30140680 PMC6095029

[2] Collins AM. Advances in ileitis control, diagnosis, epidemiology and the economic impacts of disease in commercial pig herds. Agriculture. (2013) 3:536–55. 10.3390/agriculture3030536

[3] Lawson GHK Rowland AC MacIntyre N. Demonstration of a new intracellular antigen in porcine intestinal adenomatosis and hamster proliferative ileitis. Vet Microbiol. (1985) 10:303–13. 10.1016/0378-1135(85)90001-x 2412336

[4] Lawson GH McOrist S Jasni S Mackie RA. Intracellular bacteria of porcine proliferative enteropathy: cultivation and maintenance *in vitro*. J Clin Microbiol. (1993) 31:1136–42. 10.1128/jcm.31.5.1136-1142.1993 8501214 PMC262892

[5] Vannucci FA Pusterla N Mapes SM. Evidence of host adaptation in Lawsonia intracellularis infections. Vet Res. (2012) 43:53. 10.1186/1297-9716-43-53 22715937 PMC3443049

[6] Beckler DC Kapur V Gebhart CJ. Molecular epidemiologic typing of Lawsonia intracellularis.In: Proceedings of the Conference of Research Workers in Animal Disease. Chicago, IL: Ellis RP. 2004, Blackwell Publishing.

[7] Holyoake PK Collins A Donahoo M Lising R Emery D. Identifying obstacles to reducing the use of antibiotics to control porcine proliferative enteropathy. Aust Vet J. (2009) 87:33–4. 10.1111/j.1751-0813.2008.00372.x 19178474

[8] Opriessnig T Karuppannan AK Beckler D Ait-Ali T Cubas-Atienzar A Halbur PG. Bacillus pumilus probiotic feed supplementation mitigates *Lawsonia intracellularis* shedding and lesions. Vet Res. (2019) 50:85. 10.1186/s13567-019-0696-1 31640784 PMC6806562

[9] Muwonge A Karuppannan AK Opriessnig T. Probiotics mediated gut microbiota diversity shifts are associated with reduction in histopathology and shedding of Lawsonia intracellularis. Anim Microbiome. (2021) 3:22. 10.1186/s42523-021-00084-6 33663618 PMC7931366

[10] Vannucci FA Foster DN Gebhart CJ. Comparative transcriptional analysis of homologous pathogenic and non-pathogenic Lawsonia intracellularis isolates in infected porcine cells. PLoS ONE. (2012) 7:e46708. 10.1371/journal.pone.0046708 23056413 PMC3463550

[11] Leite FL Abrahante JE Vasquez E Vannucci F Gebhart CJ Winkelman N. Cell Proliferation and Inflammatory Signature Is Induced by Lawsonia intracellularis Infection in Swine. mBio. (2019) 10:e01605–18. 10.1128/mBio.01605-18 30696739 PMC6355989

[12] Huan YW Bengtsson RJ MacIntyre N Guthrie J Finlayson H Smith SH. Lawsonia intracellularis exploits β-catenin/Wnt and Notch signalling pathways during infection of intestinal crypt to alter cell homeostasis and promote cell proliferation. PLoS ONE. (2017) 12:e0173782. 10.1371/journal.pone.0173782 28323899 PMC5360247

[13] Salvesen HA Sargison FA Archibald AL Ait-Ali T. Characterisation of autophagy disruption in the ileum of pigs infected with Lawsonia intracellularis. Vet Res Commun. (2022) 46:585–92. 10.1007/s11259-021-09847-7 34669106 PMC9165227

[14] Yang L Lai F He L Lu Y Zhong Q Lai C. LI1035, a putative effector secreted by Lawsonia intracellularis, targets the MAPK pathway and regulates actin organizationin yeast and mammalian cells. Vet Microbiol. (2019) 235:127–35. 10.1016/j.vetmic.2019.06.009 31282370

